# Comparative Transcriptome Analysis of Salivary Glands of Two Populations of Rice Brown Planthopper, *Nilaparvata lugens*, That Differ in Virulence

**DOI:** 10.1371/journal.pone.0079612

**Published:** 2013-11-14

**Authors:** Rui Ji, Haixin Yu, Qiang Fu, Hongdan Chen, Wenfeng Ye, Shaohui Li, Yonggen Lou

**Affiliations:** 1 State Key Laboratory of Rice Biology, Institute of Insect Science, Zhejiang University, Hangzhou, China; 2 Research and Development Center of Rice Production Technology, China National Rice Research Institute, Hangzhou, China; 3 State Key Laboratory of Rice Biology, Institute of Biotechnology, Zhejiang University, Hangzhou, China; Natural Resources Canada, Canada

## Abstract

**Background:**

The brown planthopper (BPH), *Nilaparvata lugens* (Stål), a destructive rice pest in Asia, can quickly overcome rice resistance by evolving new virulent populations. Herbivore saliva plays an important role in plant–herbivore interactions, including in plant defense and herbivore virulence. However, thus far little is known about BPH saliva at the molecular level, especially its role in virulence and BPH–rice interaction.

**Methodology/Principal Findings:**

Using cDNA amplification in combination with Illumina short-read sequencing technology, we sequenced the salivary-gland transcriptomes of two BPH populations with different virulence; the populations were derived from rice variety TN1 (TN1 population) and Mudgo (M population). In total, 37,666 and 38,451 unigenes were generated from the salivary glands of these populations, respectively. When combined, a total of 43,312 unigenes were obtained, about 18 times more than the number of expressed sequence tags previously identified from these glands. Gene ontology annotations and KEGG orthology classifications indicated that genes related to metabolism, binding and transport were significantly active in the salivary glands. A total of 352 genes were predicted to encode secretory proteins, and some might play important roles in BPH feeding and BPH–rice interactions. Comparative analysis of the transcriptomes of the two populations revealed that the genes related to ‘metabolism,’ ‘digestion and absorption,’ and ‘salivary secretion’ might be associated with virulence. Moreover, 67 genes encoding putative secreted proteins were differentially expressed between the two populations, suggesting these genes may contribute to the change in virulence.

**Conclusions/Significance:**

This study was the first to compare the salivary-gland transcriptomes of two BPH populations having different virulence traits and to find genes that may be related to this difference. Our data provide a rich molecular resource for future functional studies on salivary glands and will be useful for elucidating the molecular mechanisms underlying BPH feeding and virulence differences.

## Introduction

Insect herbivore saliva contains digestive enzymes, such as alkaline phosphatase, esterase, amylase and β-glucosidase, as well as other components, such as elicitors that induce plant defense, effectors that inhibit plant defense, and proteins related to pathogen transmission [1]–[3]. Some studies have also found a relationship between saliva components and herbivore virulence [4]. Therefore, herbivore saliva, the first substance to come into chemical contact with the plant, plays important roles in both food ingestion and interactions between herbivores and their host plants [1]–[5]. Characterizing herbivore saliva will provide new insights into plant–herbivore interactions, including induced plant defense and herbivore virulence.

To characterize herbivore saliva, the transcriptome and/or proteome of the salivary glands and/or saliva of several herbivore species – mostly hemipterans such as rice brown planthopper (BPH; *Nilaparvata lugens* (Stål)); Hemiptera: Delphacidae) [6], [7], pea aphid (*Acyrthosiphon pisum*; Hemiptera: Aphididae) [8], [9], [10], green peach aphid (*Myzus persicae*; Hemiptera: Aphididae) [11], [12], whitefly (*Bemisia tabaci* (Gennadius); Hemiptera: Aleyrodidae) [13], and potato leafhopper (*Empoasca fabae* (Harris); Hemiptera: Cicadellidae) [14] – have been analyzed. These studies found several hundred proteins in the saliva [4], [6]–[14]. However, whether differences in salivary components exist between herbivore populations with different virulence traits and what functions these components have remain largely unanswered questions.

BPH, one of the most destructive insect pests of the rice plant (*Oryza sativa* L.) in Asia, causes substantial losses of rice yield every year by sucking phloem sap and transmitting plant viruses, such as the rice ragged stunt virus and the rice grassy stunt virus [15]. The cultivation of resistant rice varieties is an important control measure for the BPH. However, the BPH rapidly overcomes rice resistance by evolving new virulent populations [16]. BPH virulence strains generally correspond to particular resistance genes in rice. For example, rice varieties TN1 (a susceptible variety) and Mudgo (carrying the resistance gene *bph1*) host virulent BPH strains 1 and 2, respectively [16]. Although some minor differences in morphology and in the composition of bacterial symbionts among virulence genotypes have been reported, the mechanisms underlying changes in BPH virulence are not clear [16]–[19].

When sucking sap, BPHs and other phloem feeders secrete two primary kinds of saliva: coagulable and watery saliva. Coagulable saliva forms salivary sheaths, which help to stabilize the insects' stylets and suppress plant defense responses to components of the watery saliva [1], [4]. Watery saliva, which contains a mixture of amino acids, proteins, and digestive enzymes, assists the movement of the stylets inside the salivary sheath, the digestion of plant material, and the suppression of plant defense responses [1]–[4], [9], [12]. Given the important roles of herbivore saliva in plant-herbivore interactions, BPH saliva is likely to play a central role in the interaction between BPHs and rice, including in the evolution of BPH virulence.

Salivary gland morphology and several salivary proteins of BPHs have been reported [20]–[24]. Moreover, Noda *et al*. (2008) identified 2383 expressed sequence tags (ESTs) in the salivary glands of the BPH by Sanger’s method [6]. However, these data are insufficient to elucidate the nature of BPH saliva. Moreover, whether BPH populations with different virulence have different saliva components remains unclear. To explore these issues, we compared the salivary-gland transcriptomes of two BPH populations with different virulence that were maintained on one of two rice varieties: TN1 (TN1 population) or Mudgo (M population). Using next-generation high-throughput sequencing technology, a total of 37,666 and 38,451 unigenes were identified in the respective populations, and 3,757 unigenes were differentially expressed between the two populations. Moreover, among the differentially expressed genes, 67 unigenes encoded putative secretory proteins. These findings provide an exciting opportunity to elucidate the components of BPH saliva and suggest a possible correlation between BPH saliva and virulence.

## Results and Discussion

### Illumina Sequencing and Read Assembly

The cDNA libraries of the salivary glands of BPH TN1 and M populations were sequenced using an Illumina platform, resulting in 38,487,746 and 40,350,780 reads, respectively. After cleaning and quality checks, short sequences were assembled into 97,103 and 101,885 contigs, respectively. Using paired end-joining and gap-filling methods, these contigs were further assembled into 73,510 and 76,195 scaffolds, which were then clustered into 37,666 and 38,451 unigenes, respectively (Table 1). Finally, sequence data from the two libraries were combined to obtain 43,312 unigenes with a mean length of 746 bp. The length distributions of the unigenes were similar between the two populations, suggesting there was no bias in the cDNA library construction (Figure S1). However, some unigenes were found in only one population (data not shown). We believe these differences may have been caused by differential long-term ecological adaptations to the different rice hosts. The total number of unigenes obtained was much higher than the number of BPH salivary gland ESTs identified by Noda *et al.* [6], indicating the depth of coverage possible using HiSeq technology.

**Table 1:** Summary statistics for salivary gland transcriptomes of two brown planthopper populations.

	TN1 population	M population
Total number of raw reads	38,487,746	40,350,780
Total number of clean reads	35,948,162	37,583,138
Total length of clean reads	3,235,334,580	3,382,482,420
Total number of contigs	97,103	101,885
Mean length of contigs	268	265
GC percentage	45.42%	45.28%
Total number of scaffolds	73,510	76,195
Total unique sequences	37,666	38,451
Mean length of unique sequences	599	609

### Annotation of Salivary-gland Transcripts

**Figure 1:**
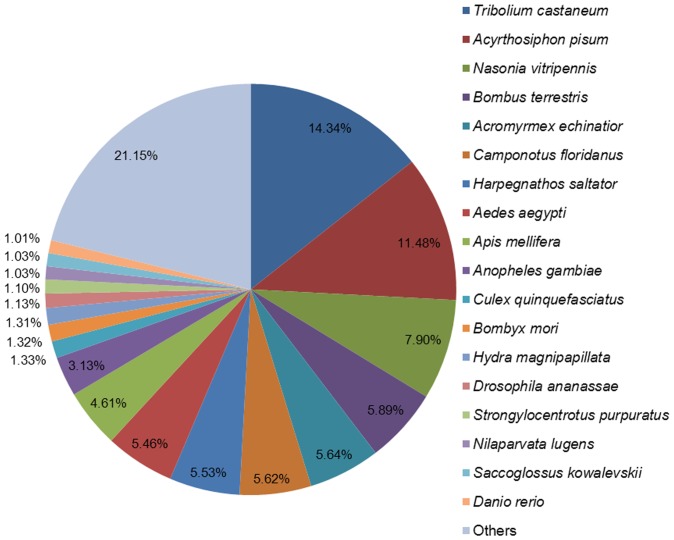
Species distribution of the BLASTX results of brown planthopper salivary-gland genes. This figure shows the species distribution of unigene BLASTX results against the NCBI nr protein database with a cutoff E-value ≤1.0E^–5^ and the proportions of each species, represented by different colors. Species with proportions greater than 1% are shown.

For functional annotation, the 43,312 unigenes were searched using BLASTx against the non-redundant (nr) NCBI protein database. A total of 19,771 unigenes (45.65% of all distinct sequences) yielded significant BLAST hits with a cut-off E-value of 1.0E^–5^ (Table S1). The species distributions of the best matches for each sequence are shown in Figure 1. The highest percentages of unique sequences matched genes of the red flour beetle (*Tribolium castaneum*; 14.34%), *A. pisum* (11.48%), and a parasitoid wasp (*Nasonia vitripennis*; 7.90%). A similar result was also reported by Xue et al. [25], Peng et al. [26] and Bao et al. [27]. Further research should explore why the highest percentage of unique sequences matches genes of *T. castaneum*, a coleoptera beetle, rather than *A. pisum*, a hemiptera aphid which is much more closely related to BPH.

### GO and KEGG Orthology Classifications

**Figure 2:**
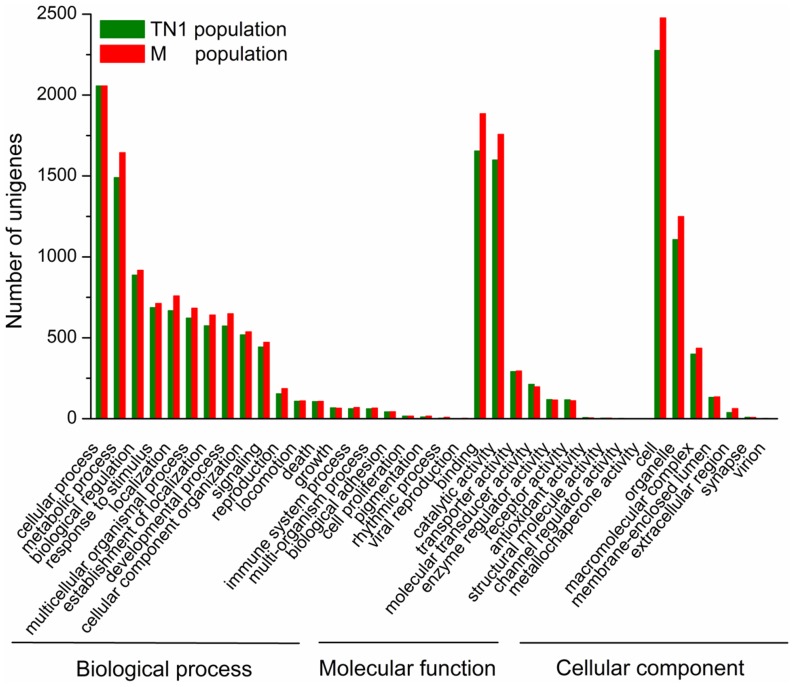
Gene ontology (GO) classifications of salivary-gland genes of TN1 and Mudgo (M) populations. The results are summarized in three main categories: biological process, molecular function, and cellular component. The *y*-axis shows the number of matching unigenes in a category.

Gene Ontology (GO) assignments were used to functionally classify the predicted proteins. A total of 18,500 sequences from TN1 population and 17,118 from M population were categorized into 38 functional groups (Figure 2). GO has three ontologies: ‘biological process,’ ‘molecular function,’ and ‘cellular component.’ The results of GO analysis showed a similar distribution of gene functions between the two populations (Figure 2). Among both populations, the three dominant basic ‘biological process’ categories are ‘cellular process,’ ‘metabolic process,’ and ‘biological regulation.’ These results indicated that the cells in the salivary glands were metabolically active, which correlated well with the biological function of the tissue of interest. In ‘molecular function,’ the three most abundant categories are ‘binding,’ ‘catalytic activity,’ and ‘transporter activity.’ This ‘binding’ category includes sequences annotated to be involved in protein, nucleic acid, ion, cofactor and enzyme binding. Saliva secretion is the main function of the salivary glands, and saliva is composed of water, electrolytes, lipids, amino acids and proteins. Not surprisingly, a large number of genes we found are involved in binding and transport. In ‘cell component’, the top three are ‘cell’, ‘organelle’ and ‘macromolecular complex’. This suggests that cells and organelles are the prominent parts of the salivary glands, and macromolecular complex secretion is also critical in the salivary glands.

**Figure 3:**
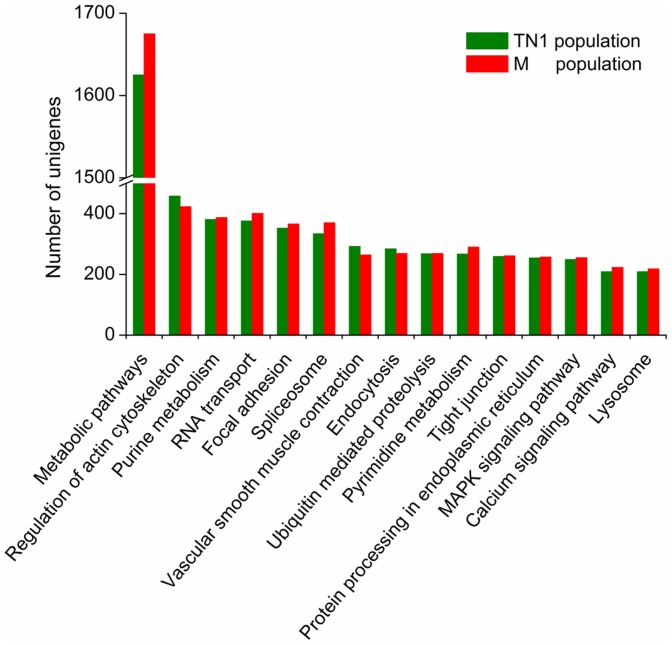
KEGG pathway distributions of unigenes from salivary glands of TN1 and Mudgo (M) populations. The top fifteen pathways (excluding human disease-related) with the highest percentages of mapped unigenes are shown.

To investigate which biological pathways are active in the salivary glands, all of the sequences were assigned to the reference canonical pathways in the Kyoto Encyclopedia of Genes and Genomes (KEGG). A similar distribution of biological pathways for TN1 and M populations was also found by KEGG mapping, and a total of 11449 unigenes from TN1 population and 11502 unigenes from M population were mapped separately to 239 and 240 pathways in total (Tables S2 and S3). The salivary glands are specific organs for salivary macromolecule production and, consequently, have a high level of metabolic activity. This characteristic was implied in a previous study by observing the dense cytoplasm and organized whorls of rough endoplasmic reticulum in the salivary-gland cells [28]. Consequently, among these pathways, “metabolism” is important in the salivary glands (1625 unigenes from TN1 population and 1675 unigenes from M population, Figure 3, in pathways associated with human diseases were excluded). Since there are abundant proteins in the saliva of BPHs, as a major original location of these proteins, the salivary glands should be active in protein synthesis and catabolism. Indeed, the transcriptome contained many sequences involved in the “protein processing in endoplasmic reticulum” pathway (254 genes in TN1 population and 257 genes in M population), which is related to the formation and transport of nascent secretory proteins, and the ‘lysosome’ pathway (209 genes in TN1 population and 218 genes in M population), a major pathway related to protein degradation. The GO annotations and KEGG orthology (KO) classifications indicated that the salivary glands might be active in metabolism, binding and transport, and that a high percentage of genes might be involved in secretory protein processing and secretion.

### Unigenes Encoding Putative Secreted Proteins

We characterized the salivary secretome of the BPH by combining the transcripts of the salivary glands of the two populations. Secreted proteins are probably delivered into saliva through the eukaryotic endoplasmic reticulum–Golgi pathway [29]. Proteins secreted through this pathway have an N-terminal signal peptide. Therefore, all amino acid sequences were analyzed for the presence of signal peptides and potential cleavage sites. A total of 464 unique genes encoded a secretory signal peptide. Of these, 112 were predicted to have at least one transmembrane domain besides the signal peptide, indicating that their proteins were likely embedded in cell membranes of the salivary glands. Thus, 352 potential secretory proteins were retained (Table S4), which is comparable to the numbers of putative secreted proteins reported in *A. pisum* (324) and *B. tabaci* (295) [10], [13]. Interestingly, the possible functions of some putative secreted proteins were closely related to the known roles of insect saliva, such as digestion and suppressing or eliciting plant defenses.

Among the putative secreted proteins in the BPH, a set of digestive enzymes and hydrolases, including plant cell wall (PCW)-degrading enzymes were found. These putative PCW-degrading enzymes included one β-1,4-endoglucanase (Unigene1860_All), one β-glucosidase (Unigene26172_All), and two β-1,3-glucanases (Unigene10762_All and Unigene23029_All). PCW, a thick, rigid polysaccharide structure comprising an extensive network of celluloses, hemicelluloses and pectins, forms a formidable barrier to herbivores. To overcome this barrier, herbivores have evolved to secrete salivary PCW-degrading enzymes, such as cellulases (consisting of endoglucanases and β-glucosidases) and pectinases [1], [29], [30]. For example, an extract of *Homalodisca vitripennis* salivary gland contained a variety of enzymes capable of hydrolyzing glycosidic linkages in the polysaccharides of PCWs, including β-1,4-endoglucanase, xyloglucanase and β-1,4-endoxylanase; these enzymes could digest xyloglucans in cell walls to facilitate *H. vitripennis* stylet penetration and were related to the formation of the salivary sheath [31]. In addition to degrading cellulose cooperating with endoglucanases, salivary β-glucosidases in the B- and D-follicles of BPHs act on phenolic glucosides, such as arbutin and salicin [24], [30]. It has been reported that β-1,3-glucanases secreted by plant pathogens can degrade β-1,3-glucans in callose [32]. Callose has been reported to exist in the PCWs of a wide variety of higher plants and can be induced by either biotic or abiotic stress, including BPH feeding [33], [34]. Callose deposition plays an important role in preventing BPHs from ingesting phloem sap [34]. Thus β-1,3-glucanases in BPH saliva may play a role in countering rice defenses by inhibiting the formation and deposition of callose.

In addition to PCW-degrading enzymes, other digestive enzymes and hydrolases found in putative secreted proteins in BPH salivary glands included 11 trypsin-like proteins or serine proteases (Table S5), 3 glycosyl hydrolases [α-amylase (Unigene9457_All), sucrase (Unigene8043_All) and α-glucosidase (Unigene3598_All)], 2 lipases (Unigene18263_All and Unigene41792_All) and a phospholipase A_2_ (Unigene23547_All). In the plant phloem sap, there are many proteins (including defense-related proteins), sugars, amino acids, organic acids, ATP and so on [35], [36]. Therefore, these putative secreted proteins, like PCW-degrading enzymes, may function in extra-oral digestion and/or the suppression of defenses. Salivary α-glucosidase in the G-follicle of the principal gland of the BPH, for example, hydrolyzes sucrose and trehalose [24]. α-Amylases aid in pre-digesting starch to maltose by hydrolyzing α-D-(1,4)-glucan bonds; the maltose is then hydrolyzed to glucose by an α-glucosidase [37]–[40]. A recent study showed that a salivary lipase (MdesL1) of *Mayetiola destructor* was involved in extra-oral digestion and host cell permeability [41]. In another study, phospholipase A_2_ in the salivary glands of *Manduca sexta* may have been involved in the hydrolysis of fatty acid moieties from dietary phospholipids and also in killing food-borne bacteria [42]. The functional characterization of these hydrolases and of the digestive enzymes of the BPH may shed light on this species' mode of extra-oral digestion and plant defense suppression.

Of the putative secreted proteins, we also identified 3 odorant-binding proteins (OBPs) and 9 chemosensory proteins (CSPs) (Table S6), and all nine putative CSPs showed high homology to MP10 (E-value ≤1.0E^–5^), a salivary effector found in aphids [43]. OBPs and CBPs were generally reported to be preferentially expressed in antennae, mouthparts, and other chemosensory organs [44]–[47], and to be involved in perireceptor events of the chemosensory system. Because these proteins transport chemical signals to sensory neuron receptors, they can be said to regulate insect behavior, such as communication among conspecifics, predator detection, and food/host location [45], [48]. Recently, some members of OBPs and CSPs were also found to be broadly expressed in various insect tissues, including salivary glands; in addition, their functions are involved in many areas, such as nymph survival [49], development of embryonic integuments [50], insect leg regeneration [51], and insect-host interactions [43], [52], [53]. CSPs, for example, were the most abundant proteins in *Vanessa cardui* and *V. gonerilla* mandibular gland lumens, and they were thought to play an important role in host plant recognition, helping the insect perceive phagostimulants and deterrents, and in the detection of microorganisms on the leaf surface [52]. Some OBPs in mosquito saliva that are predicted to function in olfaction and gustation are also secreted into host cells to manipulate host physiology by scavenging host amines [53]. Of the three identified salivary effectors – C002, Mp42 and Mp10, in aphids – Mp10, homologous to a CSP, induced chlorosis and local cell death in *Nicotiana benthamiana* and suppressed the oxidative burst induced by flg22 [43]. This suggests that the putative secreted CSPs and OBPs found in BPH salivary glands may also play a role in BPH-rice interactions, an issue that is worthy of future study.

As a consequence of co-evolution, BPH saliva may contain some components to counter plant secondary chemicals. In the BPH salivary secretome, we found five putative secreted carboxylesterases (Unigene3085_All, Unigene12962_All, Unigene13873_All, Unigene14160_All, and Unigene35902_All). It has been well documented that carboxylesterases in insect midguts and fat body tissues play important roles in metabolizing xenobiotics (*i.e.*, plant toxic allelochemicals and synthetic chemicals, such as pesticides) [27], [54]–[58]. In plant phloem sap, there are also many defense chemicals [36], [59]. Thus, these putative secreted carboxylesterases in BPH salivary glands might also be related to detoxification.

Interestingly, alkaline phosphatase (ALP) (Unigene1963_All) was identified in the salivary secretome; a ubiquitous enzyme in all organisms, it is characterized by its ability to hydrolyze orthophosphate monoesters at alkaline pH [60]. Evidence suggests that insect ALPs are expressed in various tissues, and are involved in absorption and transport, development, neural and renal function, cuticle sclerotization and xenobiotic tolerance [61]–[64]. ALPs have also been detected in the salivary glands of several insects, and their activity was confirmed in the saliva of wheat aphids (*Diuraphis noxia*) and *B. tabaci* Biotype B [65]–[68]. Salivary ALPs have been suggested to be involved in producing the stylet sheath, and thus they may aid in plant feeding [67], [68]. This possible role of salivary ALPs in the BPH will require further investigation.

In addition to aiding in digestion and suppressing plant defense, these putative proteins secreted by salivary glands may also be recognized by plants, thus eliciting plant defense responses. Thus far, some of these enzymes and/or their products, such as lipase and β-glucosidase, have been reported to elicit signaling pathways that activate both direct and indirect plant defense responses [69]–[72]. The lipase activity in *Schistocerca gregaria* oral secretions, for example, plays a central role in the accumulation of oxylipins, especially 12-oxo-phytodienoic acid [70]. In BPH saliva, β-glucosidase was also found to induce salicylic acid-, H_2_O_2_- and ethylene-mediated signaling pathways [71], [72]. It is expected that ongoing efforts will point to more such elicitors in herbivore saliva.

Although many homologs of insect secretory proteins were found in the BPH salivary secretome, some were not identified. For example, the pectinase complex, comprising endopolygalacturonases, rhamnogalacturonan lyases and pectin methylesterases, plays an important role in pectin degradation, which subsequently facilitates the decomposition of (hemi)cellulose and makes the PCWs more susceptible to further breakdown by other enzymes [29], [69], [73]. Pectinases occur mainly in Hemiptera and Coleoptera [69]. However, in the BPH salivary secretome, pectinases were not identified. This suggests that BPH may use unique feeding strategies. Alternatively, these enzymes may have been filtered out during the prediction of secretory proteins because they have incomplete 5′ sequences. In our experiments, for instance, the unigene Unigene1860_All was annotated to encode a cellulase. However, because it lacks the 5′ end, this unigene was filtered out. Therefore, to completely explore the protein composition of BPH saliva, other research approaches, such as the proteome analysis of BPH saliva, should also be used.

### Differences in Salivary-gland Gene Expression Profiles between TN1 and M Populations

**Figure 4:**
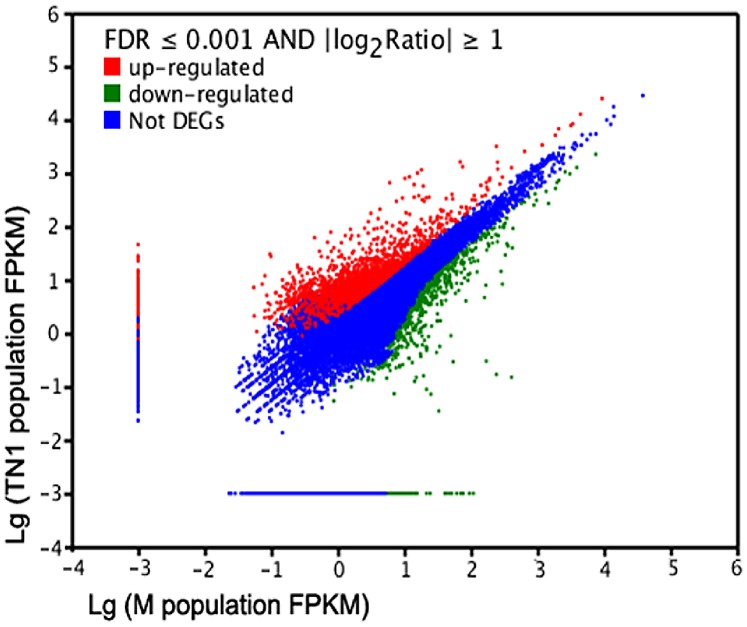
Bitmap of differentially expressed genes. The red and green points represent up- and down-regulated genes in the salivary glands of TN1 population, respectively; the blue points represent genes with no differences in expression based on the criteria of the false discovery rate (FDR) ≤0.001 and an absolute value of the log_2_ ratio ≥1.

**Figure 5:**
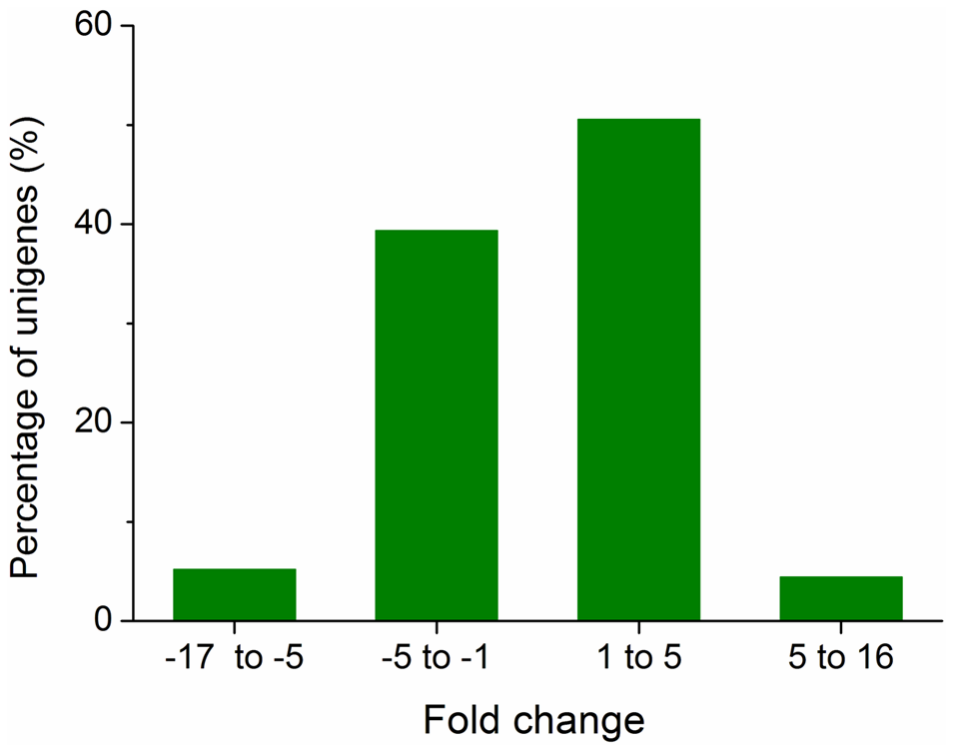
Fold difference distribution of differently expressed genes. The *x*-axis shows the fold change (log_2_ ratio) in gene expression in TN1 population compared to Mudgo (M) population.

Gene expression levels can be estimated from Illumina sequencing data based on the number of raw reads [74]. To identify differentially expressed genes in the salivary glands of TN1 and M populations, the number of fragments mapped to each gene was calculated and normalized using fragments per kb per million fragments (FPKM) [74], [75]. We found 3,757 genes with significantly different expression levels (Figure 4, Table S7). Of these, 2,084 were up-regulated in TN1 population relative to M population. The detected fold differences (log_2_ ratio) ranged from −17 to +16, and 89.88% were up- or down-regulated by from 1.0- to 5.0-fold (Figure 5). To validate these gene expression data, we compared the expression profiles of salivary glands of TN1 and M populations using quantitative real-time polymerase chain reaction (QRT-PCR). Of the 21 randomly selected genes, 20 showed concordant fold differences between the types of analyses (Table S8), indicating that our results were reliable.

**Figure 6:**
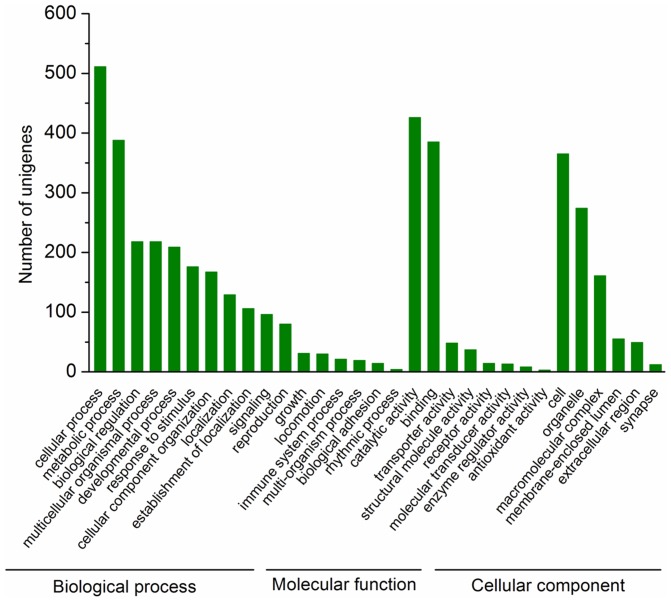
Distribution of significantly differentially expressed transcripts in gene ontology (GO) subclasses. The *y-*axis shows the number of the matching differentially expressed genes in that category.

The differentially expressed transcripts were assigned to 31 functional groups, such as ‘biological process,’ ‘molecular function,’ and ‘cellular component’; ontology distributions are shown in Figure 6. Among the ‘biological process’ assignments, many genes were assigned to ‘cellular process,’ ‘metabolism process,’ and ‘biological regulation.’ In the ‘molecular function’ category, most genes were related to ‘catalytic activity,’ ‘binding,’ and ‘transporter activity.’ ‘Cell’ formed the main part of the ‘cell component’ category. The differentially expressed genes coding for these physiological processes may affect the saliva composition of these populations; further functional studies must be performed to validate this hypothesis.

To gain insight into the dominant biological pathways of the differentially expressed genes that mapped to KO, a hypergeometric test was performed to explore statistically enriched pathways. In total, 33 enriched pathways (*P*≤0.05) were identified (Table S9; pathways associated with human disease were excluded). Ten gene sets were correlated to ‘metabolism’ and four to ‘digestion and absorption’ (Table 2). Carbohydrates, especially sucrose, are the dominant chemical component in rice phloem sap, and they are particularly important macronutrients for BPHs. Digestible carbohydrates are used primarily for energy but can also be converted to fat and stored, and their carbon skeletons can contribute to amino acid production. Low levels of carbohydrate metabolism can limit insect growth and performance. Indeed, many gene groups (72 genes) involved in ‘carbohydrate metabolism’ and ‘carbohydrate digestion and absorption’ were enriched, including ‘galactose metabolism’, ‘starch and sucrose metabolism’, and ‘carbohydrate digestion and absorption’. Of these 72 genes, 46 (63.89%) were up-regulated in the salivary glands of M population (Table 2). There are also many differentially expressed genes related to ‘metabolism’ and ‘digestion and absorption’ of other nutrients and substances, such as α-linolenic acid, glycerolipid, amino acids, fat, vitamins, and proteins (Table 2). Interestingly, the ‘salivary secretion’ pathway including 30 genes was also enriched; of these genes, 20 genes (66.67%) were up-regulated in the salivary glands of M population (Table S9). Salivary secretion plays an important role in plant-insect interactions. This analysis indicates that the difference in ‘metabolism’, ‘digestion and absorption’ and ‘salivary secretion’ may contribute to the change in virulence between the two populations. However, more evidence is required to evaluate this hypothesis.

**Table 2:** Differentially-expressed genes related to ‘metabolism’ and ‘digestion and absorption’ pathways.

KEGG Pathway	*P*-value	Total^1^	Up-regulated^2^	Down-regulated^3^
**etabolism**				
Alpha-linolenic acid metabolism	6.76E-05	14	8	6
Glycerolipid metabolism	4.89E-04	26	12	14
Amino sugar and nucleotide sugar metabolism	1.98E-03	24	21	3
Starch and sucrose metabolism	3.01E-03	30	17	13
Pyrimidine metabolism	3.15E-03	52	15	37
Retinol metabolism	4.24E-03	15	5	10
Galactose metabolism	4.56E-03	22	14	8
Glycerophospholipid metabolism	5.26E-03	27	18	9
Drug metabolism - cytochrome P450	1.76E-02	12	4	8
Linoleic acid metabolism	4.41E-02	5	4	1
**Digestion and absorption**				
Fat digestion and absorption	8.13E-07	29	13	16
Vitamin digestion and absorption	1.29E-05	25	7	18
Protein digestion and absorption	8.98E-03	39	15	24
Carbohydrate digestion and absorption	1.14E-02	20	15	5

1 Number of differentially expressed genes in salivary glands belonging to each KEGG pathway.

2 Number of genes up-regulated in brown planthopper M population relative to TN1 population in each KEGG pathway.

3 Number of genes down-regulated in brown planthopper M population relative to TN1 population in each KEGG pathway.

Among the genes encoding putative secreted proteins of BPH salivary glands, 67 were found to be differentially expressed between the BPH populations. Of these, 43 genes were up-regulated, while 24 were down-regulated in M population compared to TN1 population (Table S10). The detected fold differences (log_2_ ratio) ranged from –6.82 to +4.86, and 79.10% of the genes were up- or down-regulated by from 1.0- to 3.0-fold (Table S10). As stated above, many proteins in herbivore saliva play important roles in herbivore feeding and herbivore-plant interactions; thus these differentially expressed putative secreted proteins might mediate BPH virulence.

## Conclusions

This study provides the first comprehensive evaluation of the salivary gland transcriptome of BPH using high-throughput sequencing and comparative expression analysis between two BPH populations, TN1 and M, with different virulence. In total, we obtained 43,312 unique sequences and 3,757 differentially expressed genes. GO and KO analysis indicate that genes related to metabolism, binding and transport were significantly active in the salivary glands. A total of 352 genes were predicted to encode secretory proteins, and some might play important roles in BPH feeding and BPH-rice interactions. Comparative analysis of the transcriptomes of the two populations revealed that the genes related to ‘metabolism,’ ‘digestion and absorption,’ and ‘salivary secretion’ might be associated with virulence traits. Furthermore, some putative secretory proteins that were differentially expressed in the two populations were identified. These findings provide a valuable resource for future investigations of the composition of BPH saliva and the role of saliva in changes in BPH virulence and interactions between BPH and rice.

## Materials and Methods

### BPH Cultures, Salivary-gland Collection, and RNA Isolation

Two different virulent populations of BPH, TN1 and M, were maintained on rice varieties TN1 and Mudgo, respectively, for more than 170 generations at 27±1°C and 70±10% relative humidity under a 14/10 h light/dark photoperiod. The original insects were provided by the Chinese National Rice Research Institute (Hangzhou, China). BPH adult females, within 2 days after eclosion, were placed in a Petri dish on ice, and their salivary glands were dissected in phosphate buffer saline (pH 7.2) using forceps. For RNA extraction, the salivary glands of about 150 females were pooled for each population and directly dipped in RNA Lysis Buffer (Promega, Fitchburg, WI, USA). Total RNA was isolated using the SV Total RNA Isolation System (Promega) according to the manufacturer's protocol. The concentration and quality of total RNA were determined by a NanoDrop spectrophotometer (Thermo Fisher, Waltham, MA, USA).

### Library Construction of the Salivary Glands and Illumina Sequencing

The salivary-gland cDNA library was prepared using a SMARTer™ PCR cDNA Synthesis Kit (Clontech, Mountain View, CA, USA) and an Advantage 2 PCR Kit (Clontech) following the kit’s instructions. After end repair and adaptor ligation, the products were amplified by PCR and purified using QIAquick PCR Purification Kit (Qiagen, Hilden, Germany). This cDNA library was then sequenced on a Hiseq2000 Illumina sequencing platform in BGI-Shenzhen (Shenzhen, China). Raw reads were generated using a Solexa GA Pipeline 1.6 (Illumina). After the removal of empty and low-quality reads with undetermined nucleotides (‘N’), processed reads were assembled using SOAPdenovo software and clustered with TGI Clustering tools [76], [77]. All raw transcriptome data was deposited in the NCBI short read archive (SRA) with accession number SRX276865 (TN1 population) and SRX276866 (M population). The assembled sequences whose orientations were predicted via blast or ESTScan (recorded in 5′ to 3′) have been deposited in the NCBI transcriptome shotgun assembly (TSA) database with accession number 214044 TSA. The generated unigenes were screened with BLAST (http://blast.ncbi.nlm.nih.gov) and annotated against the nr and SwissProt databases with an E-value cut-off of 1.0E^–5^. GO and KO annotations of the unigenes were determined using BLAST2GO (http://www.blast2go.org/) and Inter-ProScan software [13], [25].

### Secretory Protein Prediction

To translate the unigene nucleotide sequences into amino acid sequences, we first searched all the combined transcriptome unigenes against protein databases using BLASTx (E-value ≤1.0E^–5^, switch -F F) in the following order – nr, Swiss-Prot, KEGG, and clusters of orthologous groups (COG) – until the sequences had significant hits. The BLAST results were used to extract coding sequences (CDSs). The CDSs of unigenes with no significant BLAST hits were predicted by ESTScan [13]. We then used the SignalP 4.0 Server (http://www.cbs.dtu.dk/services/SignalP/) to predict the presence of signal peptides and cleavage sites in the amino acid sequences. To predict transmembrane domains, we submitted each amino acid sequence with a signal peptide to the TMHMM Server v. 2.0 (http://www.cbs.dtu.dk/services/TMHMM/) [13], [43]. Putative proteins with a signal peptide and 0–1 transmembrane domain (the signal peptide can be a transmembrane domain) were considered to be potential secreted proteins [13].

### Analysis of Differential Gene Expression

Salivary gland genes that were differentially expressed between TN1 and M populations were identified using a table of counts constructed with FPKM values, which adjusted the number of fragments by the total number of fragments mapped and the length of the gene [74], [75]. The false discovery rate (FDR) was used to determine threshold *P*-values in multiple test and analysis. An FDR<0.001 and an absolute value of the log_2_ ratio >1 provided significance thresholds for gene expression differences.

### QRT-PCR Analysis

To confirm the results of FPKM analysis, the expression levels of 21 randomly selected salivary-gland genes were measured in TN1 and M populations by QRT-PCR. Total RNA from each sample (salivary glands of about 120 females per sample) was extracted using the SV Total RNA Isolation System. For each total RNA sample, 0.75 µg of RNA was reverse-transcribed using the PrimeScript RT–PCR Kit (TaKaRa, Otsu, Japan). QRT-PCR was carried out in a CFX96TM Real-Time System (Bio-Rad, Hercules, CA, USA) using iQ™ SYBR Green (Bio-Rad) under the following conditions: 95°C for 3 min, then 40 cycles of 95°C for 5 s, 60°C for 15 s, and melting curve generation from 65 to 95°C. Primers used for QRT–PCR are listed in Table S11. Each gene was analyzed in three biological replications, after which the average threshold cycle (C_t_) per sample was calculated. The endogenous actin gene was used for normalization. A no-template control sample (using nuclease-free water) was run to detect contamination and to determine the degree of dimer formation (data not shown). Finally, relative expression levels were calculated as 2^–ΔΔCt^.

### Identification of Statistically Enriched KEGG Pathways

The differentially expressed genes were used for KO enrichment analysis using the hypergeometric test to measure significantly enriched terms. The formula was:
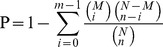



In this equation, *N* indicates the number of genes with KO annotations and *n* the number of differentially expressed genes in *N*. The variables *M* and *m* represent the numbers of genes and of differentially expressed genes, respectively, in each KO term. The threshold to determine significant enrichment of the gene sets was corrected to *P*-value ≤0.05.

## Supporting Information

Figure S1 **Length distribution of unigenes in salivary-gland transcriptomes of brown planthopper populations.** The *x-*axis shows the calculated lengths of unigenes in the salivary-gland library and the *y*-axis shows the number of unigenes. (A) TN1 population. (B) M population.
(TIF)

Table S1 **Top BLASTX hits from NCBI non-redundant (nr) database.** All unigenes were screened using BLASTX against the NCBI nr database with a cut-off E-value ≤1.0E^–5^.
(XLSX)

Table S2 **KEGG orthology (KO) annotation of unigenes from the salivary-gland transcriptome of TN1 population of the brown planthopper (BPH).**
(XLSX)

Table S3 **KEGG orthology (KO) annotation of unigenes from the salivary-gland transcriptome of Mudgo (M) population of the brown planthopper (BPH).**
(XLSX)

Table S4 **Genes encoding putative secreted proteins.** Based on the coding sequences of translated unigenes, the presence of a signal peptide was predicted using SignalP v. 4.0, and cleavage position was calculated, resulting in 464 proteins with putative secretion signals. Transmembrane domains in these proteins were predicted with the TMHMM Server v. 2.0. After removing sequences for proteins that were likely embedded in cell membranes, 352 predicted secretory proteins were retained. They were annotated by searching against the nr, Swiss-Prot, and COG databases.
(XLSX)

Table S5 **Genes encoding putative secreted trypsin-like proteins and serine proteases.**
(XLSX)

Table S6 **Genes encoding putative secreted odorant-binding and chemosensory proteins.**
(XLSX)

Table S7 **Differentially expressed genes indicated by the FPKM statistical analysis.** FPKM, FDR-value, fold change (log_2_ ratio) of gene expression, and best hits against nr (E-value ≤1.0E^–5^) for all gene pairs are listed in this table.
(XLSX)

Table S8 **Gene expression data verified by quantitative real-time polymerase chain reaction (QRT-PCR).** Twenty-one unigenes were selected for expression-level validation using QRT-PCR.
(XLSX)

Table S9 **Statistically significantly enriched KEGG pathways.** Human disease-associated pathways were excluded from this analysis.
(XLSX)

Table S10 **Differentially expressed genes encoding putative secreted proteins indicated by FPKM values.**
(XLSX)

Table S11 **Primers used in quantitative real-time polymerase chain reaction (QRT-PCR) for validation of differentially expressed genes.**
(XLSX)

## References

[1] Miles PW (1999) Aphid saliva. Biol Rev 74: 41–85.

[2] DE Vos M, Jander G (2009) *Myzus persicae* (green peach aphid) salivary components induce defence responses in *Arabidopsis thaliana* . Plant Cell Environ 32: 1548–1560. 19558622 10.1111/j.1365-3040.2009.02019.x

[3] Hogenhout SA, Bos JIB (2011) Effector proteins that modulate plant-insect interactions. Curr Opin Plant Biol 14: 422–428. 21684190 10.1016/j.pbi.2011.05.003

[4] Nicholson SJ, Hartson SD, Puterka GJ (2012) Proteomic analysis of secreted saliva from Russian Wheat Aphid (*Diuraphis noxia* Kurd.) biotypes that differ in virulence to wheat. J Proteomics 75: 2252–2268. 22348819 10.1016/j.jprot.2012.01.031

[5] Will T, Tjallingii WF, Thonnessen A, van Bel AJE (2007) Molecular sabotage of plant defense by aphid saliva. Proc Natl Acad Sci USA 104: 10536–10541. 17553961 10.1073/pnas.0703535104 PMC1965548

[6] Noda H, Kawai S, Koizumi Y, Matsui K, Zhang Q, et al (2008) Annotated ESTs from various tissues of the brown planthopper *Nilaparvata lugens*: a genomic resource for studying agricultural pests. BMC Genomics 9: 117. 18315884 10.1186/1471-2164-9-117 PMC2311293

[7] Konishi H, Noda H, Tamura Y, Hattori M (2009) Proteomic analysis of the salivary glands of the rice brown planthopper, *Nilaparvata lugens* (Stål) (Homoptera: Delphacidae). Appl Entomol Zool 44: 525–534.

[8] Mutti NS (2006) Molecular studies of the salivary glands of the pea aphid, *Acyrthosiphon pisum*. Ph.D. thesis, Kansas State University.

[9] Carolan JC, Fitzroy CIJ, Ashton PD, Douglas AE, Wilkinson TL (2009) The secreted salivary proteome of the pea aphid *Acyrthosiphon pisum* characterised by mass spectrometry. Proteomics 9: 2457–2467. 19402045 10.1002/pmic.200800692

[10] Carolan JC, Caragea D, Reardon KT, Mutti NS, Dittmer N, et al (2011) Predicted effector molecules in the salivary secretome of the pea aphid (*Acyrthosiphon pisum*): A dual transcriptomic/proteomic approach. J Proteome Res 10: 1505–1518. 21226539 10.1021/pr100881q

[11] Ramsey J, Wilson A, De Vos M, Sun Q, Tamborindeguy C, et al (2007) Genomic resources for *Myzus persicae*: EST sequencing, SNP identification, and microarray design. BMC Genomics 8: 423. 18021414 10.1186/1471-2164-8-423 PMC2213679

[12] Harmel N, Létocart E, Cherqui A, Giordanengo P, Mazzucchelli G, et al (2008) Identification of aphid salivary proteins: a proteomic investigation of *Myzus persicae* . Insect Mol Biol 17: 165–174. 18353105 10.1111/j.1365-2583.2008.00790.x

[13] Su YL, Li JM, Li M, Luan JB, Ye XD, et al (2012) Transcriptomic analysis of the salivary glands of an invasive whitefly. PLoS ONE 7: e39303. 22745728 10.1371/journal.pone.0039303 PMC3379992

[14] DeLay B, Mamidala P, Wijeratne A, Wijeratne S, Mittapalli O, et al (2012) Transcriptome analysis of the salivary glands of potato leafhopper, *Empoasca fabae* . J Insect Physiol 58: 1626–1634. 23063500 10.1016/j.jinsphys.2012.10.002

[15] Hibino H (1996) Biology and epidemiology of rice viruses. Annu Rev Phytopathol 34: 249–274. 15012543 10.1146/annurev.phyto.34.1.249

[16] Claridge MF, Hollander JD (1980) The “biotypes” of the rice brown planthopper, *Nilaparvata lugens* . Entomol Exp Appl 27: 23–30.

[17] Claridge MF, Hollander JD, Haslam D (1984) The significance of morphometric and fecundity differences between the “biotypes” of the brown planthopper, *Nilaparvata lugens* . Entomol Exp Appl 36: 107–114.

[18] Hollander JD, Pathak PK (1981) The genetics of the “biotypes” of the rice brown planthopper, *Nilaparvata lugens* . Ent Exp Appl 29: 76–86.

[19] Tang M, Lav L, Jing SL, Zhu LL, He GC (2010) Bacterial symbionts of the brown planthopper. Appl Environ Microbiol 76: 1740–1745. 20097822 10.1128/AEM.02240-09 PMC2838002

[20] Sogawa K (1965) Studies on the salivary glands of rice plant leafhoppers. I. Morphology and histology. Jpn J Appl Entomol Zool 19: 275–290.

[21] Sogawa K (1967a) Chemical nature of the sheath materials secreted by leafhoppers (Homoptera). Appl Entomol Zool 2: 13–21.

[22] Sogawa K (1967b) Studies on the salivary glands of rice plant leafhoppers. II. Origins of the structural precursors of the sheath material. Appl Entomol Zool 2: 195–202.

[23] Sogawa K (1968a) Studies of the salivary glands of rice leafhoppers. III. Salivary phenolase. Appl Entomol Zool 3: 13–25.

[24] Sogawa K (1968b) Studies on the salivary glands of rice plant leafhoppers. IV. Carbohydrate activities. Appl Entomol Zool 3: 67–73.

[25] Xue J, Bao YY, Li BL, Cheng YB, Peng ZY, et al (2010) Transcriptome analysis of the brown planthopper *Nilaparvata lugens* . PLoS One 5: e14233. 21151909 10.1371/journal.pone.0014233 PMC2997790

[26] Peng X, Zha W, He R, Lu T, Zhu L, et al (2011) Pyrosequencing the midgut transcriptome of the brown planthopper, *Nilaparvata lugens* . Insect Mol Biol 20: 745–762. 21919985 10.1111/j.1365-2583.2011.01104.x

[27] Bao YY, Wang Y, Wu WJ, Zhao D, Xue J, et al (2012) *De novo* intestine-specific transcriptome of the brown planthopper *Nilaparvata lugens* revealed potential functions in digestion, detoxification and immune response. Genomics 99: 256–264. 22361737 10.1016/j.ygeno.2012.02.002

[28] Ghanim M, Rosell RC, Campbell LR, Czosnek H, Brown JK, et al (2001) Digestive, salivary, and reproductive organs of *Bemisia tabaci* (Gennadius) (Hemiptera: Aleyrodidae) B type. J Morphol 248: 22–40. 11268056 10.1002/jmor.1018

[29] Cherqui A, Tjallingii WF (2000) Salivary proteins of aphids, a pilot study on identification, separation and immunolocalisation. J Insect Physiol 46: 1177–1186. 10818245 10.1016/s0022-1910(00)00037-8

[30] Watanabe H, Tokuda G (2010) Cellulolytic systems in insects. Annu Rev Entomol 55: 609–632. 19754245 10.1146/annurev-ento-112408-085319

[31] Backus EA, Andrews KB, Shugart HJ, Greve CL, Labavitch JM, et al (2012) Salivary enzymes are injected into xylem by the glassy-winged sharpshooter, a vector of *Xylella fastidiosa* . J Insect Physiol 58: 949–959. 22587965 10.1016/j.jinsphys.2012.04.011

[32] Tenberge KB, Brockmann B, Tudzynski P (1999) Immunogold localization of an extracellular β-1,3-glucanase of the ergot fungus *Claviceps purpurea* during infection of rye. Mycol Res 103: 1103–1118.

[33] Jacobs AK, Lipka V, Burton RA, Panstruga R, Strizhov N, et al (2003) An *Arabidopsis* callose synthase, GSL5, is required for wound and papillary callose formation. Plant Cell 15: 2503–2513. 14555698 10.1105/tpc.016097 PMC280557

[34] Hao P, Liu CX, Wang YY, Chen RZ, Tang M, et al (2008) Herbivore-induced callose deposition on the sieve plates of rice: an important mechanism for host resistance. Plant Physiol 146: 1810–1820. 18245456 10.1104/pp.107.111484 PMC2287352

[35] Hayashi H, Fukuda A, Suzui N, Fujimaki S (2000) Proteins in the sieve element-companion cell complexes: their detection, localization and possible functions. Funct Plant Biol 27: 489–496.

[36] Kehr J (2006) Phloem sap proteins: their identities and potential roles in the interaction between plants and phloem-feeding insects. J Exp Bot 57: 767–774. 16495410 10.1093/jxb/erj087

[37] Grossman G, James A (1993) The salivary glands of the vector mosquito, *Aedes aegypti*, express a novel member of the amylase gene family. Insect Mol Biol 11: 223–232. 10.1111/j.1365-2583.1993.tb00095.x 7505701

[38] Feng GH, Richardson M, Chen MS, Kramer KJ, Morgan TD, et al (1996) Alpha-amylase inhibitors from wheat: amino acid sequences and patterns of inhibition of insect and human alpha-amylases. Insect Biochem Mol Biol 26: 419–426. 8763161 10.1016/0965-1748(95)00087-9

[39] Ohashi K, Natori S, Kubo T (1999) Expression of amylase and glucose oxidase in the hypopharyngeal gland with an age-dependent role change of the worker honeybee (*Apis mellifera* L.). Eur J Biochem 265: 127–133. 10491166 10.1046/j.1432-1327.1999.00696.x

[40] Ngernyuang N, Kobayashi I, Promboon A, Ratanapo S, Tamura T, et al (2011) Cloning and expression analysis of the *Bombyx mori* α-amylase gene (*Amy*) from the indigenous Thai silkworm strain, Nanglai. J Insect Sci 11: 38. 21529256 10.1673/031.011.0138 PMC3281462

[41] Shukle RH, Mittapalli O, Morton PK, Chen MS (2009) Characterization and expression analysis of a gene encoding a secreted lipase-like protein expressed in the salivary glands of the larval Hessian fly, *Mayetiola destructor* (Say). J Insect Physiol 55: 104–111. 19026654 10.1016/j.jinsphys.2008.10.008

[42] Tunaz H, Stanley DW (2004) Phospholipase A2 in salivary glands isolated from tobacco hornworms, *Manduca sexta* . Comp Biochem Phys B 139: 27–33. 10.1016/j.cbpc.2004.05.010 15364285

[43] Bos JIB, Prince D, Pitino M, Maffei ME, Win J, et al (2010) A functional genomics approach identifies candidate effectors from the aphid species *Myzus persicae* (green peach aphid). PLoS Genet 6: e1001216. 21124944 10.1371/journal.pgen.1001216 PMC2987835

[44] Jacobs SP, Liggins AP, Zhou JJ, Pickett JA, Jin X, et al (2005) OS-D-like genes and their expression in aphids (Hemiptera: Aphididae). Insect Mol Biol 14: 423–432. 16033435 10.1111/j.1365-2583.2005.00573.x

[45] Xu YL, He P, Zhang L, Fang SQ, Dong SL, et al (2009) Large-scale identification of odorant-binding proteins and chemosensory proteins from expressed sequence tags in insects. BMC Genomics 10: 632. 20034407 10.1186/1471-2164-10-632 PMC2808328

[46] Pelletier J, Leal WS (2011) Characterization of olfactory genes in the antennae of the southern house mosquito, *Culex quinquefasciatus* . J Insect Physiol 57: 915–929. 21504749 10.1016/j.jinsphys.2011.04.003

[47] Iovinella I, Dani FR, Niccolini A, Sagona S, Michelucci E, et al (2011) Differential expression of odorant-binding proteins in the mandibular glands of the honey bee according to caste and age. J Proteome Res 10: 3439–3449. 21707107 10.1021/pr2000754

[48] Whiteman N, Pierce N (2008) Delicious poison: genetics of Drosophila host plant preference. Trends Ecol Evol 23: 473–478. 18657878 10.1016/j.tree.2008.05.010

[49] He P, Zhang J, Liu NY, Zhang YN, Yang K, et al (2011) Distinct expression profiles and different functions of odorant binding proteins in *Nilaparvata lugens* Stål. PLoS ONE 6: e28921. 22174925 10.1371/journal.pone.0028921 PMC3235172

[50] Maleszka J, Forêt S, Saint R, Maleszka R (2007) RNAi-induced phenotypes suggest a novel role for a chemosensory protein CSP5 in the development of embryonic integument in the honeybee (*Apis mellifera*). Dev Genes Evol 217: 189–196. 17216269 10.1007/s00427-006-0127-y

[51] Nomura A, Kawasaki K, Kubo T, Natori S (1992) Purification and localization of p10, a novel protein that increases in nymph regenerating legs of *Periplaneta americana* (American cockroach). Int J Dev Biol 36: 391–398. 1445782

[52] Celorio-Mancera MP, Sundmalm SM, Vogel H, Rutishauser D, Ytterberg AJ, et al (2012) Chemosensory proteins, major salivary factors in caterpillar mandibular glands. Insect Biochem Mol Biol 42: 796–805. 22885177 10.1016/j.ibmb.2012.07.008

[53] Calvo E, Mans BJ, Andersen JF, Ribeiro JM (2005) Function and evolution of a mosquito salivary protein family. J Biol Chem 281: 1935–1942. 16301315 10.1074/jbc.M510359200

[54] Després L, David JP, Gallet C (2007) The evolutionary ecology of insect resistance to plant chemicals. Trends Ecol Evol 22: 298–307. 17324485 10.1016/j.tree.2007.02.010

[55] Ross MK, Streit TM, Herring KL, Xie S (2012) Carboxylesterases: dual roles in lipid and pesticide metabolism. J Pestic Sci 35: 257–264. 10.1584/jpestics.R10-07 PMC4087164 25018661

[56] Pauchet Y, Wilkinson P, van Munster M, Augustin S, Pauron D, et al (2009) Pyrosequencing of the midgut transcriptome of the poplar leaf beetle *Chrysomela tremulae* reveals new gene families in Coleoptera. Insect Biochem Mol Biol 39: 403–413. 19364528 10.1016/j.ibmb.2009.04.001

[57] Karatolos N, Pauchet Y, Wilkinson P, Chauhan R, Denholm I, et al (2011) Pyrosequencing the transcriptome of the greenhouse whitefly, *Trialeurodes vaporariorum* reveals multiple transcripts encoding insecticide targets and detoxifying enzymes. BMC Genomics 12: 56. 21261962 10.1186/1471-2164-12-56 PMC3036619

[58] Ramsey JS, Rider DS, Walsh TK, De Vos M, Gordon KHJ, et al (2010) Comparative analysis of detoxification enzymes in *Acyrthosiphon pisum* and *Myzus persicae* . Insect Mol Biol 19: 155–164. 20482647 10.1111/j.1365-2583.2009.00973.x

[59] Givovich A, Sandstrom J, Niemeyer HM, Pettersson J (1994) Presence of a hydroxamic acid glucoside in wheat phloem sap, and its consequences for performance of *Rhopalosiphum padi* (L) (Homoptera, Aphididae). J Chem Ecol 20: 1923–1930. 24242719 10.1007/BF02066233

[60] Eguchi M (1995) Alkaline phosphatase isozymes in insects and comparison with mammalian enzyme. Comp Biochem Phys B 111: 151–162. 10.1016/0305-0491(94)00248-s 7599983

[61] Yan Y, Peng L, Liu WX, Wan FH, Harris MK (2011) Host plant effects on alkaline phosphatase activity in the whiteflies, *Bemisia tabaci* Biotype B and *Trialeurodes vaporariorum* . J Insect Sci 11: 9. 21521136 10.1673/031.011.0109 PMC3281299

[62] Nathan SS (2006) Effects of *Melia azedarach* on nutritional physiology and enzyme activities of the rice leaffolder *Cnaphalocrocis medinalis* (Guenée) (Lepidoptera: Pyralidae). Pestic Biochem Phys 84: 98–108.

[63] Nathan SS, Choi MY, Paik CH, Seo HY (2007) Food consumption, utilization, and detoxification enzyme activity of the rice leaffolder larvae after treatment with *Dysoxylum triterpenes* . Pestic Biochem Phys 88: 260–267.

[64] Wang ZX, Liu HH, Yang BJ, Liu ZW (2011) Characterization of soluble and membrane-bound alkaline phosphatase in *Nilaparvata lugens* and their potential relation to development and insecticide resistance. Arch Insect Biochem 78: 30–45. 10.1002/arch.20437 21769927

[65] Srivastava JP, Saxena SC (1967) On the alkaline and acid phosphatase in the alimentary tract of *Periplaneta americana* L. (Blattaria: Blattidae) Appl Entomol Zool. 2: 85–92.

[66] Kumar D, Ray A, Ramanurt PS (1980) Studies on the salivary gland of *Lygaeus* sp. (Lygaeidae: Heteroptera) - histological, histochemical, autoradiographic and electron microscopic investigations. Z Mikrosk Anat Forsch 4: 669–695. 6161493

[67] Copper WR, Dillwith JW, Puterka GJ (2010) Salivary proteins of Russian Wheat Aphid (Hemiptera: Aphididae). Environ Entomol 39: 223–231. 20146860 10.1603/EN09079

[68] Funk CJ (2001) Alkaline phosphatase activity in whitefly salivary glands and saliva. Arch Insect Biochem 46: 165–174. 10.1002/arch.1026 11304750

[69] Calderón-Cortés N, Quesada M, Watanabe H, Cano-Camacho H, Oyama K (2012) Endogenous plant cell wall digestion: a key mechanism in insect evolution. Annu Rev Ecol Evol S 43: 45–71.

[70] Schäfer M, Fischer C, Meldau S, Seebald E, Oelmüller R, et al (2011) Lipase activity in insect oral secretions mediates defense responses in *Arabidopsis* . Plant Physiol 156: 1520–1534. 21546453 10.1104/pp.111.173567 PMC3135923

[71] Mattiacci L, Dicke M, Posthumus MA (1995) β-glucosidase, an elicitor of herbivore-induced plant odor that attracts host-searching parasitic wasps. Proc Natl Acad Sci USA 92: 2036–2040. 11607516 10.1073/pnas.92.6.2036 PMC42418

[72] Wang X (2006) Influence of infestation by herbivores with different feeding habits or treatment by β-glucosidase on levels of main defense-related signals in rice plants. MSc Thesis, Zhejiang University.

[73] Zhang MJ, Su RX, Qi W, He ZM (2002) Enhanced enzymatic hydrolysis of lignocellulose by optimizing enzyme complexes. Appl Biochem Biotechnol 160: 1407–1414. 10.1007/s12010-009-8602-3 19288067

[74] Mortazavi A, Williams BA, McCue K, Schaeffer L, Wold B (2008) Mapping and quantifying mammalian transcriptomes by RNA-Seq. Nat Methods 5: 621–628. 18516045 10.1038/nmeth.1226 PMC13303166

[75] Audic S, Claverie JM (1997) The significance of digital gene expression profiles. Genome Res 7: 986–995. 9331369 10.1101/gr.7.10.986

[76] Li RQ, Zhu HM, Ruan J, Qian WB, Fang XD, et al (2010) De novo assembly of human genomes with massively parallel short read sequencing. Genome Res 20: 265–272. 20019144 10.1101/gr.097261.109 PMC2813482

[77] Pertea G, Huang X, Liang F, Antonescu V, Sultana R, et al (2003) TIGR Gene Indices clustering tools (TGICL): a software system for fast clustering of large EST datasets. Bioinformatics 19: 651–652. 12651724 10.1093/bioinformatics/btg034

