# Supplementary figures and images for: Comparative Transcriptome Analysis of Salivary Glands of Two Populations of Rice Brown Planthopper, Nilaparvata lugens, That Differ in Virulence
Source: PLoS One. 2013 Nov 14;8(11):e79612. doi: 10.1371/journal.pone.0079612 (PMC3828371; doi:10.1371/journal.pone.0079612)

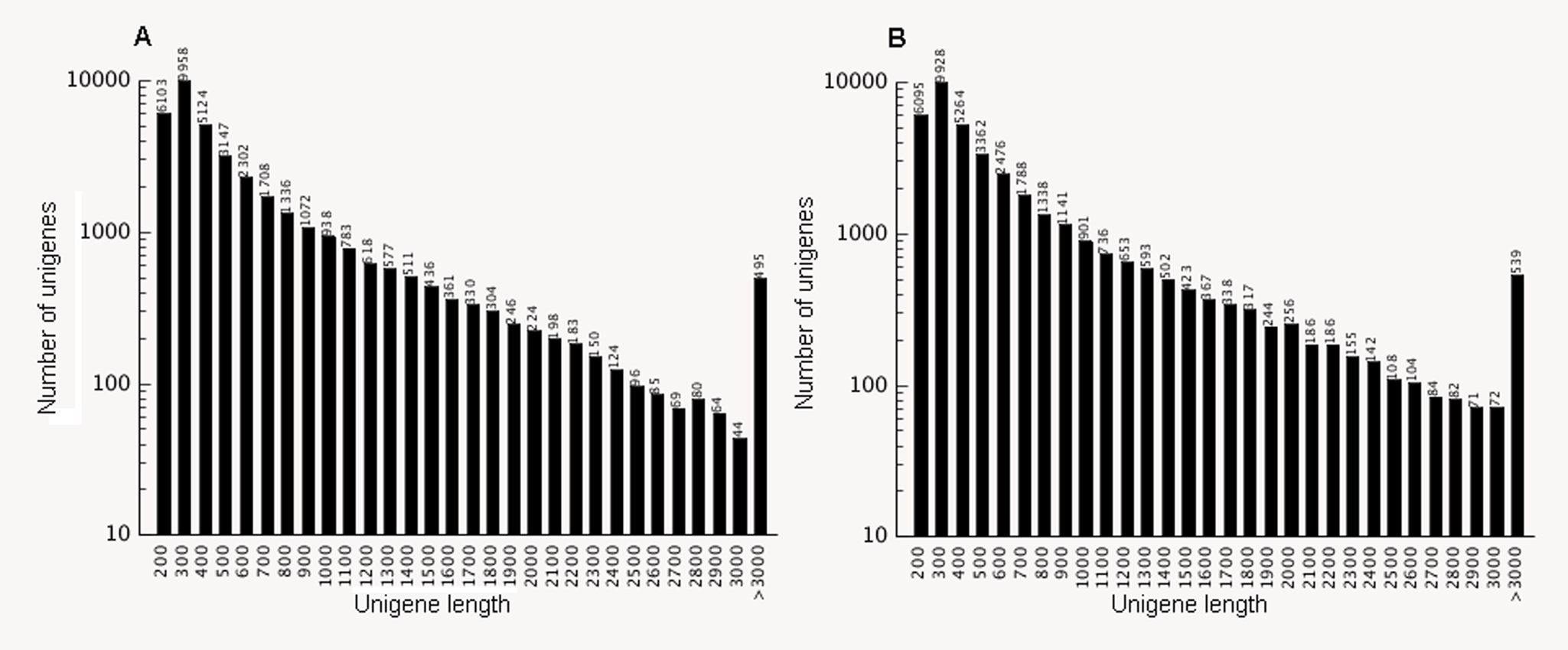

Supplement: Figure S1 — Length distribution of unigenes in salivary-gland transcriptomes of brown planthopper populations. The x-axis shows the calculated lengths of unigenes in the salivary-gland library and the y-axis shows the number of unigenes. (A) TN1 population. (B) M population. (TIF) [file pone.0079612.s001.tif]
